# Multi-disciplinary surgical approach to the management of patients with renal cell carcinoma with venous tumor thrombus: 15 year experience and lessons learned

**DOI:** 10.1186/s12894-016-0157-3

**Published:** 2016-07-19

**Authors:** Bishoy A. Gayed, Ramy Youssef, Oussama Darwish, Payal Kapur, Aditya Bagrodia, James Brugarolas, Ganesh Raj, J. Michael DiMaio, Arthur Sagalowsky, Vitaly Margulis

**Affiliations:** Department of Urology, University of Texas Southwestern Medical Center, 5323 Harry Hines Blvd., Dallas, TX 75390-9110 USA; Departments of Pathology, University of Texas Southwestern Medical Center, Dallas, TX USA; Departments of Medicine and Developmental Biology, University of Texas Southwestern Medical Center, Dallas, TX USA; Departments of Cardiothoracic Surgery, University of Texas Southwestern Medical Center, Dallas, TX USA

**Keywords:** Renal cell carcinoma, IVC thrombus, Outcomes

## Abstract

**Background:**

The management of patients with renal cell carcinoma (RCC) with venous tumor thrombus (VTT) is challenging. We report our 15 year experience in the management of patients with RCC with VTT utilizing a multidisciplinary team approach, highlighting improved total and specifically Clavien III-V complication rates.

**Methods:**

We reviewed the records of 146 consecutive patients who underwent radical nephrectomy with venous thrombectomy between 1998 and 2012. Data on patient history, staging, surgical techniques, morbidity, and survival were analyzed. Additionally, complication rates between two surgical eras, 1998–2006 and 2006–2012, were assessed.

**Results:**

The study included 146 patients, 97 males (66 %), and a median age of 61 years (range, 24–83). Overall complications rate was 53 %, high grade complications (Clavien III -V) occurred in 10 % of patients. Most importantly, there was a lower incidence of overall and high grade complications (45 % and 8 %, respectively) in the last 6 years compared to the earlier surgeries included in the study (67 % and 13 % respectively) [*p* = .008 and .03, respectively). 30 day postoperative mortality was 2.7 %. 5 year overall survival (5Y- OS) and 5 year cancer specific survival (5Y- CSS) were 51 % and 40 %, respectively. Metastasis was the only independent predictor factor for CSS (HR 3.8, CI 1.9-7.6 and *p* < .001) and OS (HR 2.6, CI 1.5-4.7 and *p* = .001) in all patients.

**Conclusions:**

Our data suggest that patients with RCC and VTT can be treated safely utilizing a multidisciplinary team approach leading to a decrease in complication rates.

**Electronic supplementary material:**

The online version of this article (doi:10.1186/s12894-016-0157-3) contains supplementary material, which is available to authorized users.

## Background

In 2015, there will be nearly 62000 newly diagnosed cases of RCC and 14000 deaths due to RCC [1]. RCC has the propensity to extend into the renal vein, inferior vena cava (IVC) and up to the right atrium in up to 23 %, 10 %, and 1 % of cases, respectively [2, 3]. Refinements in clinical imaging, with CT and MRI, have improved accurate evaluation of primary tumors and the level of venous tumor thrombus (VTT) [2, 4]. Radical nephrectomy (RN) and IVC thrombectomy (IVCT) is challenging, particularly with a high VTT level [3–5].

Throughout the course of our experience, we have continued to improve our technique employing several modifications. We believe the most important modification we have made has been constantly ensuring we have the same urologic oncologists, cardiac anesthesia team, cardiac surgeons, and a dedicated cardiac scrub team at all cases. This brings familiarity to these challenging cases, which helps better manage both intraoperative and postoperative complications. Additionally, we have deferred from preforming sternotomies for high level 3 cases to avoid the morbidity of a sternotomy. With effective liver mobilization and use of pericardial windows extirpation of high level 3 is facilitated both safely and effectively. This has equated to improved patient recovery in the postoperative setting.

Herein, we review the management of RCC with VTT in the last 15 years, aiming to outline prognostic factors, outcomes, and complication rates in the context of a dedicated multidisciplinary surgical team.

## Methods

### Patient selection

Clinical data from electronic medical records of patients treated by radical nephrectomy (RN) for RCC with renal vein or IVC thrombus at our institution from January 1998 to June 2012 were retrospectively analyzed and placed into a UT Southwestern Medical Center IRB approved database. Research was carried out in compliance with the Helsinki Declaration. We did not obtain informed consent from patients, as this was a retrospective study. Relevant clinical data, pathological features, surgical techniques, hospital stay, perioperative morbidity and mortality, follow up and survival data were collected.

### Preoperative evaluation and surgical techniques

The tumors were routinely staged by using abdominal and chest CT scans and chest radiography. MRI was used for better evaluation of VTT level at the discretion of the treating physician. Bone scans were used selectively when clinically indicated. Tumor thrombus extension was classified into 4 levels: level I, extension into the renal vein; level II, extension into the infrahepatic IVC; level III, IVC extension to the level of hepatic veins but below the diaphragm; and level IV, IVC extension above the diaphragm [6].

Preoperative renal artery angioembolization was performed at the discretion of the surgeon to facilitate arterial vascular control in patients with bulky tumor thrombus, hilar adenopathy, or hypervascularity. Surgeries were managed by a multidisciplinary team which included an experienced urologic oncology surgeon, cardiothoracic surgeon and cardiac anesthesiologist, and cardiac scrub team. Trans-esophageal echocardiography was used intraoperatively by the anesthesiologist to verify the cephalad extent of the thrombus, to monitor for tumor emboli, to confirm the complete removal of VTT, and to assess hemodynamic stability. Data regarding the duration of surgery, estimated blood loss and intraoperative complications were recorded.

### Pathologic evaluation

Pathologic staging was assigned according to the 2010 TNM staging system [7]. Grading of the tumors was evaluated according to Fuhrman classification [8]. Additionally, pathological tumor size, adrenal involvement, regional lymph node (LN) involvement, tumor necrosis, histopathological cell type and the presence of sarcomatoid differentiation were recorded.

### Outcome evaluation and statistical analysis

Perioperative morbidity and mortality within the first 30 and 90 days were recorded and graded according to the Clavien-Dindo grading system [9]. Patients without metastases were routinely followed after surgery every 3 months in the first year, every 6 months in the second year and then annually. Follow up included history, physical examination, metabolic panel, liver function tests, chest x-ray and an abdominal CT scan. Bone scan, chest CT, positron emission tomography or MRI were performed when clinically indicated.

Survival time was calculated from the date of the operation to the date of last follow up or date of death. Disease recurrence was defined as local failure in the RN bed or regional LNs, or distant metastasis. Disease-free survival (DFS) was defined as the time between the date of surgery and the development of local recurrence or distant metastasis. Censored survival values represent patients who were alive without clinical evidence of disease at the last follow up. Cancer-specific survival (CSS) and overall survival (OS) were defined as the time between the date of surgery and death due to cancer (CSS) or due to any cause (OS). The following factors that could potentially affect outcomes were analyzed: age, gender, body mass index and performance status; T stage, VTT level, pathological tumor size, nodal involvement, metastasis at presentation, grade, sarcomatoid differentiation, histological subtype, fat invasion, adrenal involvement and tumor necrosis. Endpoints were CSS and OS. DFS was analyzed only in M0 patients. Finally, independent predictors of disease recurrence and cancer specific mortality were determined using multivariate Cox Regression analyses including only factors significant in univariate analyses. Statistically significant difference was set at *p* < .05. All statistical tests were performed with SPSS version 19.0.

## Results

### Clinico-pathological features

Patient demographics and clinical characteristics of the 146 patients included in the study are shown in Table 1. Hematuria and flank pain were the most common presenting symptoms (46 % and 38 % respectively). Overall, 42 (29 %) presented with distant metastases (M+), 29 patients (20 %) had LN+. and there was no significant relation between the VTT level and presence of M+ or LN+ disease (*p* = 0.3). Metastatic sites included: lungs (11 patients), liver (5 patients), bone (5 patients), adrenal (5 patients; 4 in the ipsilateral and 1 in the contralateral adrenal) and multiple sites (5 patients).

**Table 1:** Patient demographics and clinical characteristics

	All (%)	Era 1 (%)	Era 2 (%)
All patients (%)	146 (100)	64 (44)	82 (56)
Age, median (range) y	61 ± 12	57 ± 12	64 ± 11
	(24–82)	(35–83)	(24–82)
Sex			
Male	97 (66)	42 (66)	55 (67)
Female	49 (34)	22 (34)	27 (33)
Side			
Right	82 (56)	34 (53)	48 (59)
Left	64 (44)	30 (47)	34 (41)
Race or ethnic group			
Caucasian	96 (66)	41 (64)	55 (67)
Hispanic	25 (17)	15 (23)	10 (12)
Black	15 (10)	4 (6)	11 (13)
Other	10 (7)	4 (6)	6 (7)
Presenting symptoms			
Asymptomatic	22 (15)	7 (11)	15 (18)
Flank pain	56 (38)	27 (42)	29 (35)
Hematuria	67 (46)	34 (53)	33 (40)
Weight loss	43 (30)	16 (25)	27 (33)
Lower extremity swelling	13 (9)	6 (9)	7 (9)
Change in appetite	13 (9)	4 (6)	9 (11)
Feeling of fullness	9 (6)	4 (6)	5 (6)
Distended subcutaneous veins	2 (1)	0 (0)	2 (2)
DVT/PE	8 (6)	4 (6)	4 (5)
Smoking	51 (35)	14 (22)	37 (45)
BMI median	28 ± 5.6	28 ± 4.9	26 ± 6.0
(range)	(17–56)	(19–46)	(17–56)
Obese (BMI ≥ 30)	34 (23)	17 (27)	17 (21)
ECOG			
0	27 (18)	17 (27)	10 (12)
1	93 (64)	43 (67)	50 (61)
2	20 (14)	2 (3)	18 (22)
3	6 (4)	2 (3)	4 (5)
ASA			
2	51 (35)	24 (38)	27 (33)
3	73 (50)	32 (50)	41 (50)
4	22 (15)	8 (12)	14 (17)

MRI was performed in 95 (65 %) patients for better determination of VTT level. Patients with IVC thrombus had a 26 % incidence of LN+ disease versus 14 % in those with RV only thrombus (*p* = .07). The mean number of removed and positive LNs were 5 (range, 0–33) and 1 (range, 1–22); respectively. Detailed pathological features are shown in Table 2.

**Table 2:** Pathological Features of Entire Cohort

Characteristic	Total (%)	Level I (%)	Level II (%)	Level III (%)	Level IV (%)	*P* value
	146 (100)	77 (53)	48 (33)	12 (8)	9 (6)	
T stage						<0.001
T3a	75 (51)	75 (97)	0	0	0	
T3b	51 (35)	0	40 (83)	11 (92)	0	
T3c	8 (6)	0	0	0	8 (89)	
T4	12 (8)	2 (3)	8 (17)	1 (8)	1 (11)	
Grade						0.015
1	1 (1)	0	0	1 (8)	0	
2	24 (16)	17 (22)	5 (10)	1 (8)	1 (11)	
3	86 (59)	39 (51)	32 (67)	10 (84)	5 (56)	
4	35 (24)	21 (27)	1 (23)	0	3 (33)	
Path tumor size (cm)	10.2 ± 4.5	9.2 ± 4.3	11.7 ± 4.6	10 ± 2.9	12 ± 6.3	0.02
	(2–25)	(2–23)	(3.5-25)	(5.5-15)	(5–22)	
Metastasis						0.3
Absent	104 (71)	56 (73)	31 (65)	11 (92)	6 (67)	
Present	42 (29)	21 (27)	17 (35)	1 (8)	3 (33)	
LN						0.01
N0	69 (47)	30 (39)	29 (60)	5 (42)	5 (56)	
Nx	48 (33)	36 (47)	6 (13)	4 (33)	2 (22)	
N+	29 (20)	11 (14)	13 (27)	3 (25)	2 (22)	
Sarcomatoid Differentiation						0.3
Absent	127 (87)	68 (88)	39 (81)	12 (100)	8 (89)	
Present	19 (13)	9 (1)	9 (19)	0	1 (11)	
Adrenal Involvement						0.08
Absent	128 (88)	72 (94)	38 (79)	11 (92)	7 (78)	
Present	18 (12)	5 (6)	10 (21)	1 (8)	2 (22)	
Tumor Necrosis						0.4
Absent	52 (36)	32 (42)	15 (31)	3 (25)	2 (22)	
Present	94 (64)	45 (58)	33 (69)	9 (75)	7 (78)	
Fat Invasion						0.3
Absent	28 (19)	18 (23)	7 (15)	3 (25)	0 (0)	
Present	118 (81)	59 (77)	41 (85)	9 (75)	9 (100)	
Histological Subtype						0.7
Non clear cell	11 (8)	5 (6)	5 (10)	1 (8)	0 (0)	
Clear cell	135 (92)	72 (94)	43 (90)	11 (92)	9 (100)	

### Surgical intervention

Surgical parameters and postoperative hospital stay data are included in the Additional file 1: Table S1. Preoperative renal artery angioembolization was performed in 27.4 % of all patients with any venous thrombus, and in 49.3 % of patients with level 2–4 thrombi. Chevron incision was the most common approach and was performed in all patients with level III and IV VTT (with midline strenotomy in cases where cardio-pulmonary bypass was needed). IVC clamping was used in most cases of level II and III VTT. Suprahepatic control of the IVC and control of porta hepatis were gained in level III VTT. More aggressive cardiothoracic procedures were reserved for patients with level IV and 3 patients with level III VTT who were hemodynamically unstable during the initial cross-clamping of the IVC. Right heart venovenous bypass was used to assist in removal of VTT in these 3 patients. Cardio-pulmonary bypass was needed in 5 patients with level IV VTT with mean bypass, aortic cross clamping and circulatory arrest times of 124, 59 and 25 min respectively.

Mean estimated blood loss was 1.5 L and blood loss was greatest in patients with level IV VTT. The mean operative time was around 5 h and it was correlated to level of thrombus (5.5, 6 and 6.5 h in level II, III and IV; respectively). Mean hospital stay was 8.8 days (range, 1–63) and mean ICU stay was 3 days (range, 0–51). Three patients had a complicated postoperative course and required longer care.

### Peri-operative morbidity and mortality

Out of 146 patients, 4 (2.7 %) and 5 (3.4 %) patients died within 30 and 90 days after surgery, respectively. The causes of death included: pulmonary embolism, coagulopathy, bleeding and pneumonia. Complications occurred in 77 (53 %) of patients and only 15 patients (10 %) had high grade (Clavien III-V) complications (Tables 3 and 4). The most common perioperative complication was prolonged ileus (12 %). The occurrence of complications did not correlate with VTT level or other clinical parameters including patient age, performance status, smoking and preoperative renal artery embolization (*p* > 0.05). However, correlation was seen with duration of surgery (*p* = 0.04) and intraoperative blood loss (*p* = 0.016). Most importantly, there was a lower incidence of overall and high grade complications (45 % and 8 %, respectively) in the last 6 years compared to the earlier surgeries included in the study (67 % and 13 % respectively) (*p* = .008 and .03, respectively) (Table 5).

**Table 3:** Overall complications and grading according to Clavien-Dindo system

	Total (%)	Level I (%)	Level II (%)	Level III (%)	Level IV (%)	*P* value
	146 (100)	77 (52.7)	48 (32.9)	12 (8.2)	9 (6.2)	
Overall Complications	77 (53)	38 (49)	27 (56)	7 (58)	5 (56)	.86
Ileus/bowel	18 (12)	10 (13)	4 (8)	4 (33)	0 (0)	.07
DVT	5 (3)	1 (1)	1 (2)	2 (17)	1 (11)	.03
Pleural effusion	8 (6)	3 (4)	5 (10)	0	0 (0)	.4
Acute renal failure	7 (5)	4 (5)	2 (4)	0	1 (11)	.7
Coagulopathy	5 (3)	1 (1)	3 (6)	0	1 (11)	.2
PE	10 (7)	2 (3)	1 (2)	4 (33)	3 (33)	.001
Pneumonia	2 (1)	1 (1)	1 (2)	0	0 (0)	.9
Perioperative mortality	11 (8)	3 (4)	7(15)	0	1 (11)	0.1
Clavien I-II	63 (43)	33 (43)	21 (44)	7 (58)	2 (22)	.02
Clavien III-V	15 (10)	5 (6)	6 (13)	0	4 (44)

**Table 4:** Overall complications and grading according to Clavien-Dindo system by Era

	Total (%)	Era I (%)	Era II (%)	*P* value
	146 (100)	64 (44)	82 (56)	
Overall Complications	77 (53)	43 (67)	34 (42)	.002
Ileus/bowel	18 (12)	10 (16)	8 (10)	.3
DVT	5 (3)	2 (3)	3 (4)	.9
Pleural effusion	8 (6)	3 (5)	5 (6)	.7
Acute renal failure	7 (5)	4 (6)	3 (4)	.5
Coagulopathy	5 (3)	5 (8)	0 (0)	.01
PE	10 (7)	1 (2)	9 (11)	.025
Pneumonia	2 (1)	1 (2)	1 (1)	.9
Perioperative mortality	11 (8)	2 (3)	9 (11)	0.08
Clavien I-II	63 (43)	35 (55)	28 (34)	.02
Clavien III-V	15(10)	8 (13)	7 (8)

**Table 5:** Overall High Grade Complication Rate by ERA

	Surgery Era	1998-2006	2006-2012	*p* value
Overall Complication Rate		67 %	45 %	.008
High Grade Complications Clavien III - V		13 %	8 %	.030

### Oncological outcomes

**Fig. 1:**
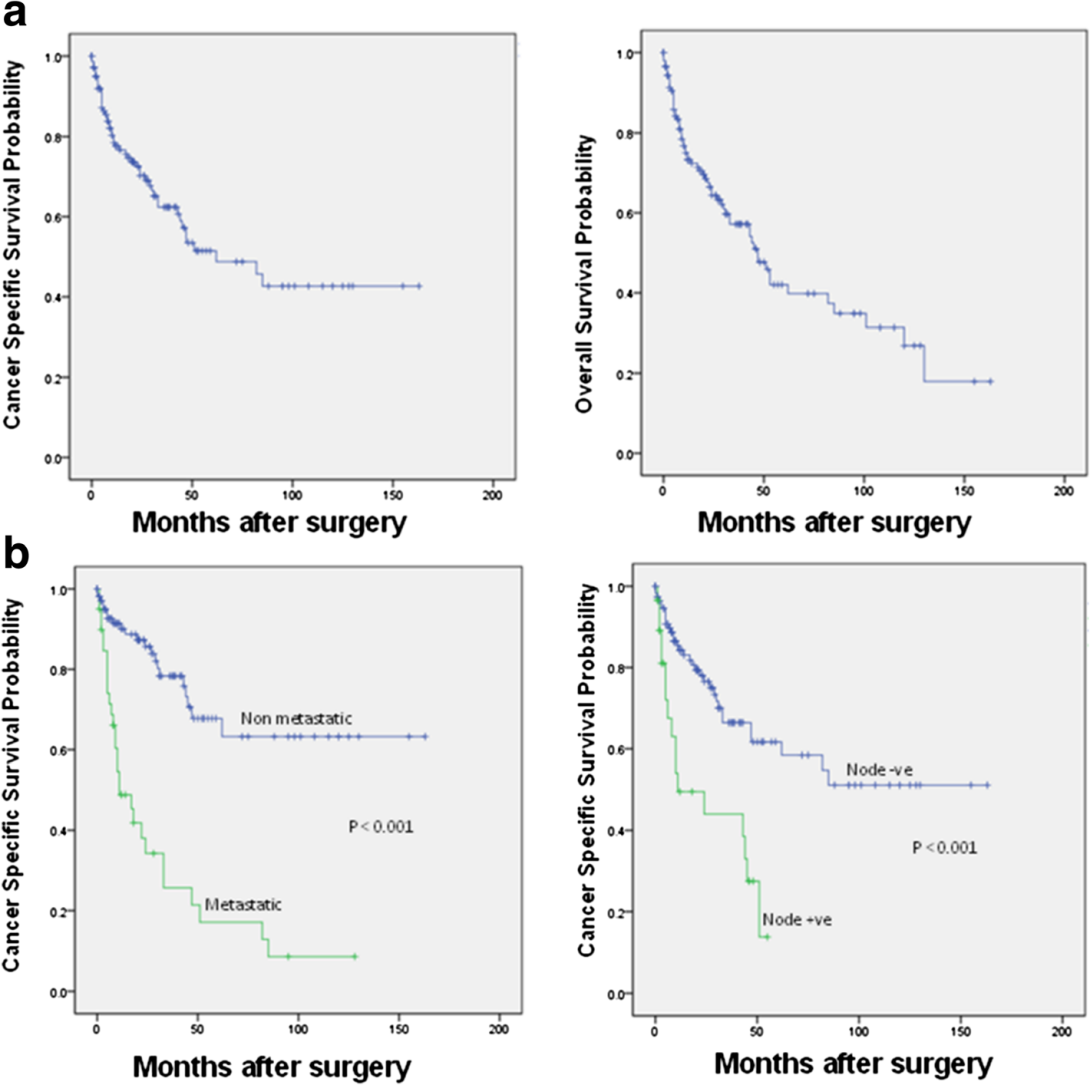
**a**) Kaplan-Meier estimates of cancer-specific survival and overall survival for 146 patients after radical nephrectomy and venous thrombectomy. **b**) Kaplan-Meier estimates of cancer-specific survival stratified by presence of metastasis at presentation and LN status for 146 patients after radical nephrectomy and venous thrombectomy

Patients were followed up after RN for a median of 16 months (mean 26, range 0–163 months). At the time of the analysis, overall mortality was 44 % with a median survival of 47 ± 4 months (range 38–56 months) and 34 % cancer specific mortality with a median CSS of 62 ± 17 months (range 29–94 months). Kaplan-Meier Survival analysis showed CSS at 2,3 and 5 years to be 70 %, 62 % and 51 %; OS at 2, 3 and 5 years to be 64 %, 57 % and 40 %; respectively (Fig. 1a). There was no significant difference in survival comparing the last 6 years to an earlier period with 3Y- CSS 62 % in both eras.

### Prognostic factors

Kaplan-Meier survival analysis (Fig. 1b) demonstrated a significant difference between CSS rates in M0 and M+ patients (5Y-CSS was 68 % and 17 % in M0 and M+ patients; respectively, *p* < 0.001). Median CSS was 11 ± 4 months for M+ patients, while it was not yet reached for M0 patients. Multivariate Cox regression analyses (data not shown) demonstrated that M+ was the only independent predictor factor for CSS (HR 3.8, CI 1.9-7.6 and *p* < .001) and OS (HR 2.6, CI 1.5-4.7 and *p* = .001). LN+ was associated with a trend toward a poor CSS (HR 1.9, CI .97-3.6 and *p* = .06). In M+ patients, LN+ was the only factor significantly associated with poor oncological outcomes as shown from CSS analysis (HR 2.3, CI 1–5 and *P* = .03), and in OS analysis (HR 2, CI .96 – 4.3 and *P* = .06). In M0 patients, high VTT level (III and IV compared to I and II) was among the independent predictors of disease recurrence (HR 4.4, CI 2.1-9.4 and *P* < .001) and cancer specific mortality (HR 6.5, CI 2–21.2; *p* = .002) in multivariate Cox regression analysis. Other independent predictors of poor oncological outcomes included larger tumor size (>13 cm) and sarcomatoid differentiation (data not shown).

## Discussion

Aggressive surgical resection is indicated in RCC with VTT as nephrectomy alone is associated with dismal prognosis [10]. Through an experienced team, consisting of a urologic oncology surgeon, cardiothoracic surgeon, cardiac anesthesiologist, and cardiac scrub team we were able to achieve satisfactory surgical and oncological outcomes and decrease the incidence of complications.

We noticed a significant reduction in the rate of overall and high grade complications (45 % and 8 %, respectively) in the last 6 years compared to the earlier surgeries included in the study (67 % and 13 % respectively) [*p* = .008 and .03, respectively]. We believe this reduction may be due to our multidisciplinary team approach, which provides uniform and consistent management of patients with VTT. The team approach further supports meticulous perioperative and postoperative planning and delivery of care, refinement of surgical technique, and improved anesthesia.

In terms of oncological outcomes, metastasis was found to be the strongest independent predictor of survival. Patients with M+ had a 3.8 times risk of cancer specific mortality compared to M0 patients (*p* < .001). While M0 patients had 5-Y CSS of 68 % and median survival that was not reached yet, M+ patients had a 17 % 5-Y CSS and 11 months median survival. Our survival rates were superior to those reported in the literature for M0 patients [3–5, 11–24] but they were similarly poor in M+ patients who were reported to have 4-30 % 5Y-CSS and 11–20 months median survival [3–5, 11–18, 21]. In this study, 29 % of patients had metastasis at presentation. This incidence was even higher in other series [14, 16]. Surgery might be indicated not only to improve oncological outcomes but also to relieve symptoms and provide better quality of life. However, performance status and associated comorbidities should be considered [10].

Overall, LN+ showed a trend toward poor CSS (HR 1.9, CI and *p* = .06) and achieved prognostic significance only in M+ patients (HR 2.3 and *p* = .03). Perhaps, statistical significance, in the analysis involving all patients, would be reached if the sample size was larger and/or follow up was longer. The independent prognostic role of LN+ was reported in other RN and IVCT series [12, 14, 21, 24]. Previous studies support the role of aggressive debulking of regional nodal disease at the time of cytoreductive RN for metastatic RCC [2, 25, 26].

There has been wide variation in reporting different prognostic factors and the prognostic value of VTT level has been debated. Our prognostic factors were similar to those reported in the largest European study that included 1192 patients from 13 European centers [12] and the US based analysis including 1875 patients with RCC and VTT from the SEER database [27]. In both studies, metastasis was the most important independent predictor of worse survival.

Interestingly, analysis of data for all patients showed that metastasis was the only independent predictor of oncological outcomes. However, in M0 patients, features associated with aggressive tumor behavior (high level VTT, large tumor size, and sarcomatoid differentiation) had an independent prognostic role. The size of the tumor has been implicated in staging of RCC. Large tumor size was among the strongest predictors of worse survival in the international RCC-VTT consortium that included 1215 RN and IVCT from 11 American and European institutions [21] as well as in a population based analysis including 1875 patients with RCC and VTT from the SEER database [27]. Sarcomatoid differentiation was reported with an incidence of 9 % and was among the independent predictors of worse survival in RN and IVCT series [3, 14, 20]. We found sarcomatoid differentiation in 13 % of tumors and it did not correlate with higher VTT levels, as 95 % of tumors with sarcomatoid differentiation had level I or II VTT.

We acknowledge several limitations in this review. First, is the retrospective design with its inherited bias. Second, while our multidisciplinary approach has lead to a decrease in the rate of complications, other factors may have also lead to improved outcomes. Improvement in surgical technique, enhanced understanding of the biology of the disease, and improved delivery of medical care throughout the course of the study may have also lead to improved patient outcomes. Lastly, the impact of venous wall invasion by thrombus could not be evaluated, as it was not reported by consistent pathologic criteria over the period under review.

## Conclusions

RN and VTT is a challenging surgery and while, improvements in surgical techniques and perioperative care have decreased surgical morbidity and mortality, we strongly advocate for management of these patients with an experienced multidisciplinary team. Our approach has resulted in improved overall complications and most importantly, high grade complications. A strong working relationship between all team members helps develop meticulous perioperative and postoperative planning and delivery of care, refinement of surgical technique, and improved anesthesia.

## Abbreviations

IVC, Inferior vena cava; IVCT, Radical nephrectomy (RN) and IVC thrombectomy; LN, Lymph node; RCC, Renal Cell Carcinoma; RN, Radical nephrectomy; VTT, Renal Cell Carcinoma (RCC) with venous tumor thrombus

## Additional file

Additional file 1: Table S1. Surgical Parameters and postoperative hospital stay. (DOCX 101 kb)
