# Supplementary material for: Multi-disciplinary surgical approach to the management of patients with renal cell carcinoma with venous tumor thrombus: 15 year experience and lessons learned
Source: BMC Urol. 2016 Jul 19;16:43. doi: 10.1186/s12894-016-0157-3 (PMC4952069; doi:10.1186/s12894-016-0157-3)
Supplement: Additional file 1: Table S1. — Surgical Parameters and postoperative hospital stay. (DOCX 101 kb) [file 12894_2016_157_MOESM1_ESM.docx]

Surgical Parameters and postoperative hospital stay

Both Eras

| **Characteristic** | **Total (%)** | **Level I (%)** | **Level II (%)** | **Level III (%)** | **Level IV (%)** |
| --- | --- | --- | --- | --- | --- |
| Patients | 146 (100) | 77 (52.7) | 48 (32.9) | 12 (8.2) | 9 (6.2) |
| Angioembolization | 40 (27) | 6 (7.8) | 21 (44) | 8 (67) | 5 (56) |
| Incision type  Transverse chevron  Midline  Flank  Thoracoabdominal  Laparoscopic | 107 (73)  9 (6)  8 (6)  12 (8)  10 (7) | 45 (59)  7 (9)  8 (10)  7 (9)  10 (13) | 41 (86)  2 (4)  0  5 (10)  0 | 12 (100)  0  0  0  0 | 9 (100)  0  0  0  0 |
| Procedure duration, min | 292 (93-688) | 204 (93-484) | 335 (199-688) | 360 (235-501) | 391 (277-549) |
| Hospital stay, days | 8.8 (1-63) | 8 (2-63) | 10 (3-51) | 9 (5-20) | 10 (1-22) |
| ICU stay, days | 3 (0-51) | 2 (0-39) | 4 (0-51) | 3 (1-7) | 4 (1-8) |
| Mean EBL, L | 1.5 (0.1-15) | 1.0 (0.1-7) | 2.0 (0.2-15) | 1.5 (0.4-3.3) | 3.7 (0.3-8.0) |
| Transfusion, units | 4.4 (0-38) | 1.9 (0-13) | 6.1 (1-24) | 2.2 (0-11) | 16.0 (1-38) |

Era 1

| **Characteristic** | **Total (%)** | **Level I (%)** | **Level II (%)** | **Level III (%)** | **Level IV (%)** |
| --- | --- | --- | --- | --- | --- |
| Patients | 64 (100) | 34 (53.1) | 25 (39.1) | 4 (6.3) | 1 (1.6) |
| Angioembolization | 18 (28) | 2 (6) | 12 (48) | 3 (75) | 1 (100) |
| Incision type  Transverse chevron  Midline  Flank  Thoracoabdominal  Laparoscopic | 42 (66)  2 (3)  2 (3)  11 (17)  7 (11) | 17 (50)  2 (6)  2 (6)  6 (18)  7 (21) | 20 (80)  0  0  5 (20)  0 | 4 (100)  0  0  0  0 | 1 (100)  0  0  0  0 |
| Procedure duration, min | - | - | - | - | - |
| Hospital stay, days | 10 (2-63) | 9 (2-63) | 10 (3-41) | 12 (5-20) | 22 (22) |
| ICU stay, days | 3 (0-39) | 3 (0-39) | 3 (0-30) | 2 (1-5) | 8 (8) |
| Mean EBL, L | 1.8 (0.1-15) | 1.0 (0.1-7) | 2.7 (0.5-15) | 1.9 (0.5-3.3) | 5.0 (5..0) |
| Transfusion, units | 6.1 (0-38) | 2.0 (0-13) | 8.8 (1-24) | 5 (0-11) | 38 (38) |

Era 2

| **Characteristic** | **Total (%)** | **Level I (%)** | **Level II (%)** | **Level III (%)** | **Level IV (%)** |
| --- | --- | --- | --- | --- | --- |
| Patients | 82 (100) | 43 (52) | 23 (28) | 8 (20) | 8 (20) |
| Angioembolization | 22 (27) | 4 (9) | 9 (39) | 5 (63) | 4 (50) |
| Incision type  Transverse chevron  Midline  Flank  Thoracoabdominal  Laparoscopic | 65 (79)  7 (9)  6 (7)  1 (1)  3 (4) | 28 (65)  5 (12)  6 (14)  1 (2)  3 (7) | 21 (86)  2 (4)  0  0  0 | 8 (100)  0  0  0  0 | 8 (100)  0  0  0  0 |
| Procedure duration, min | 292 (93-688) | 204 (93-484) | 335 (199-688) | 360 (235-501) | 391 (277-549) |
| Hospital stay, days | 8 (1-51) | 6 (2-19) | 10 (5-51) | 8 (7-10) | 9 (1-11) |
| ICU stay, days | 3 (0-51) | 4 (1-7) | 4 (3-7) | 5 (0-51) | 2 (0-9) |
| Mean EBL, L | 1.4 (0.1-15) | 1.2 (0.1-15) | 1.3 (0.2-5.5) | 1.3 (0.4-3) | 3.5 (0.3-8) |
| Transfusion, units | 3.1 (0-35) | 1.1 (0-10) | 3.4 (1-12) | 0.8 (0-4) | 15.5 (1-35) |
